# *Valeriana jatamansi* Jones ex Roxb. Against Post-Traumatic Stress Disorder, Network Pharmacological Analysis, and *In Vivo* Evaluation

**DOI:** 10.3389/fphar.2021.764548

**Published:** 2021-12-07

**Authors:** Xue Yang, Jian-You Guo, Ya-Ni Jiang, Meng-Meng Liu, Qiu-Yu Li, Jia-Yuan Li, Xiao-Jia Wei, Guo-Hui Wan, Jin-Li Shi

**Affiliations:** ^1^ School of Chinese Materia Medica, Beijing University of Chinese Medicine, Beijing, China; ^2^ CAS Key Laboratory of Mental Health, Institute of Psychology, Chinese Academy of Sciences, Beijing, China

**Keywords:** *Valeriana jatamansi* Jones ex Roxb., post-traumatic stress disorder, network pharmacology, neurotransmitters, HPA, endocannabinoid system

## Abstract

Zhi zhu xiang (ZZX) is the root and rhizome of *Valeriana jatamansi* Jones ex Roxb. Recent studies have shown that ZZX can exert antianxiety, antidepressant, and sedative effects. Because post-traumatic stress disorder (PTSD) is similar to depression and anxiety in terms of its etiology, pathogenesis, and clinical manifestations, it is possible that ZZX may also be useful for the prevention and treatment of PTSD. In this study, a mouse model of PTSD was established and used to study the pharmacological action of a 95% ethanol extract of ZZX on PTSD *via* a series of classic behavioral tests. We found that a 95% ethanol extract of ZZX was indeed effective for relieving the symptoms of PTSD in mice. Moreover, network pharmacology analysis was used to predict the potential active ingredients, targets, and possible pathways of ZZX in the treatment of PTSD. The neurotransmitter system, the hypothalamic–pituitary–adrenal (HPA) axis, and the endocannabinoid (eCB) system were identified to be the most likely pathways for anti-PTSD action in ZZX. Due to the lack of a falsification mechanism in network pharmacology, *in vivo* tests were carried out in mice, and the expression levels of neurotransmitters, hormones, and genes of key targets were detected by enzyme-linked immunosorbent assay and real-time PCR to further verify this inference. Analysis showed that the levels of norepinephrine, 5-hydroxytryptamine, and glutamic acid were increased in the hippocampus, prefrontal cortex, and amygdala of PTSD mice, while the levels of dopamine and γ-aminobutyric acid were decreased in these brain regions; furthermore, ZZX could restore the expression of these factors, at least to a certain extent. The levels of adrenocorticotropic hormone, corticosterone, and corticotropin-releasing hormone were increased in these different brain regions and the serum of PTSD mice; these effects could be reversed by ZZX to a certain extent. The expression levels of cannabinoid receptor 1 and diacylglycerol lipase α mRNA were decreased in PTSD mice, while the levels of fatty acid amide hydrolase and monoacylglycerol lipase mRNA were increased; these effects were restored by ZZX to a certain extent. In conclusion, our findings suggest that ZZX may provide new therapeutic pathways for treating PTSD by the regulation of neurotransmitters, the HPA, and expression levels of eCB-related genes in the brain.

## Introduction

Post-traumatic stress disorder (PTSD) refers to the delayed appearance and long-lasting mental disorder induced by an unusually threatening or catastrophic psychological trauma, consisting of highly heterogeneous clusters of symptoms, such as re-experience, avoidance, fear disorders, hypervigilance, anxiety, and depression (Zhang X. et al., 2021). In recent years, with the increasing number of major traumatic accidents such as wars, natural disasters, serious traffic accidents, and violent crimes, the prevalence of PTSD has been increasing year by year (Roberts et al., 2020). According to statistics, 70% of people will experience at least one traumatic event in their life and about 10–20% of individuals will suffer from PTSD (Elliott et al., 2021). The huge number of potential PTSD patients has brought pressure to society and medical treatment and has attracted social attention. Selective serotonin reuptake inhibitors (SSRIs) sertraline and paroxetine are the only FDA-approved drugs for PTSD, which use monoamine neurotransmitter systems as targets (Bandelow et al., 2012). Although these drugs have treatment effects on some symptoms of PTSD, there are still factors such as tolerance and side effects. In contrast, traditional Chinese medicine with definite curative effects and few adverse reactions has received widespread attention. Because of its multicomponent, multi-target, and multi-pathway effects, traditional Chinese medicine has highlighted its unique advantages in the prevention and treatment of PTSD (Li et al., 2020).

Zhi zhu xiang (ZZX), a traditional Chinese medicine, is derived from the roots and rhizomes of *Valeriana jatamansi* Jones ex Roxb. (Zhai et al., 2016) and has a wide history of application for relieving sleep disorders, anxiety, and depression (Biswas and Banerjee, 2018; Jugran et al., 2019). Its underground extract has long been used as a mild sedative in Europe (Toolika et al., 2015). Modern studies have shown that the 95% ethanol extract of ZZX has a high content of iridoids and has shown significant antidepressant and antianxiety activities in animal and human trials. The mechanism may be related to the regulation of neurotransmitters in the brain, improvement of HPA axis function, anti–free radical activity, anti-inflammatory property, and neuroprotection. Because PTSD is similar to depression and anxiety in its etiology, pathogenesis, and clinical manifestations, it suggests that ZZX may have a therapeutic effect on PTSD. Therefore, this research conducted a systematic study on whether the 95% ethanol extract of ZZX could alleviate the symptoms of PTSD mice and its mechanism of action, in order to provide a reference for the further development of *Valeriana jatamansi* Jones ex Roxb. medicinal resources and also a useful supplement for modern medicine to prevent and treat PTSD.

Epidemiological studies indicate that a number of factors may increase the risk of PTSD development, including neurotransmitter, hypothalamic pituitary adrenal (HPA) axis, neuroplasticity, neurotrophic factor, and the gene–environment interaction (Bazak et al., 2009; Kozlovsky et al., 2012; Li et al., 2012; Terzioğlu et al., 2013). In addition, a large number of studies have confirmed that the endocannabinoid system (ECS) is a highly specific biomarker in the pathogenesis of PTSD (Wilker et al., 2016) and an important pharmacological target for the prevention and treatment of PTSD (Neumeister et al., 2015). ECS in the brain includes cannabinoid receptors and their ligands anandamide (AEA) and 2-arachidonoylglycerol (2-AG), and related enzymes, such as fatty acid amide hydrolase (FAAH; related to AEA degradation), diacylglycerol lipase (DAGL; related to 2-AG synthesis), and monoacylglycerol lipase (MAGL; related to 2-AG degradation) (Patel et al., 2016). AEA, FAAH, and CB1 receptors are expressed in the relevant brain areas such as the basolateral amygdala (BLA), hippocampus, and medial prefrontal cortex (mPFC) that regulate stress, fear, and reward circuits (Duan et al., 2017). However, whether ZZX can treat PTSD and the potential mechanisms involved have not yet been revealed.

Network pharmacology is a new discipline that reveals the regulatory network of drugs on the body at the system level. It can predict the mechanism of drug action by constructing a complex network relationship among “drugs, active ingredients, targets, and diseases,” especially for the mechanistic prediction of multi-target drugs such as traditional Chinese medicine. This Web-based drug discovery has the advantages of economy, convenience, and reliability. However, this is only a virtual screening result, lacking a mechanism for falsification, and often requires *in vivo* or *in vitro* experimental verification to more clearly reveal the pharmacological mechanism of drug prevention and treatment of diseases (Qu et al., 2021).

**FIGURE 1:**
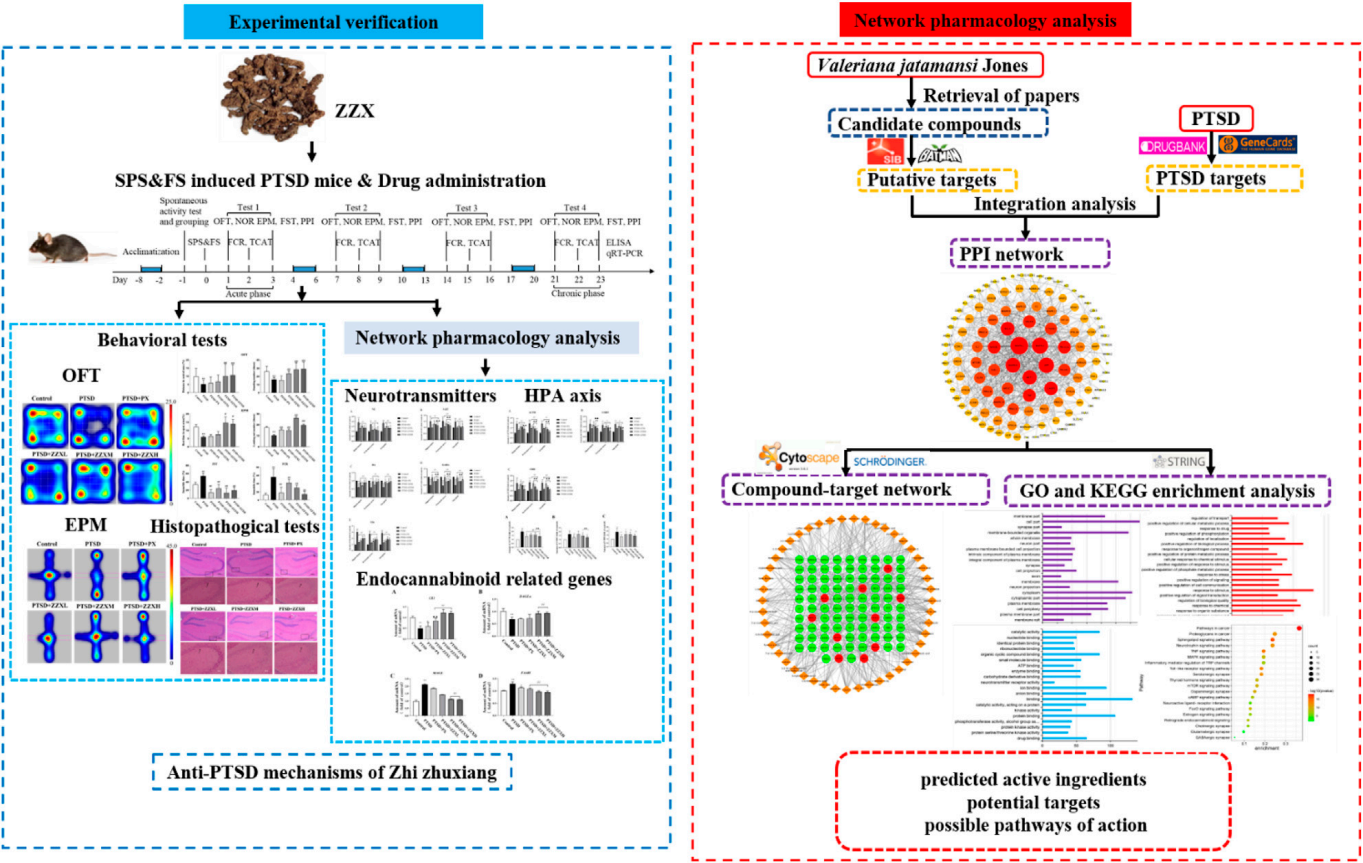
Schematic diagram of the research.

In this study, single prolonged stress (SPS) combined with foot-shock (FS) was used to establish a mouse model of PTSD. Then, we investigated the anti-PTSD efficacy of ZZX by performing a series of behavioral experiments (e.g., the open-field test) along with histopathological examinations of the hippocampus. The molecular mechanism underlying the anti-PTSD effect of ZZX was then predicted by network pharmacology. To verify the accuracy of our network pharmacology–based prediction, we performed *in vivo* tests on mice. Enzyme-linked immunosorbent assay (ELISA) and real-time PCR were then performed to detect the levels of hormones associated with the HPA axis, neurotransmitter levels, and the gene expression levels of enzymes and receptors associated with endocannabinoid (eCB) (Figure 1). This study provided a reference for the development of medical resources from *Valeriana jatamansi* Jones ex Roxb. as supplements to modern medicine for the prevention and treatment of PTSD.

## Animal Experiments

### Materials and Methods

#### Plant Material

Intact plants of *Valeriana jatamansi* Jones ex Roxb. were purchased from Yongping County Hengtong Traditional Chinese Medicine Planting Professional Cooperative (Dali Bai Autonomous Prefecture, Yunnan Province, China), and specimens of these materials (S190912) were deposited at the Department of Traditional Chinese Medicine Identification Laboratory. The plant material was authenticated by Prof. Jinli Shi. The rhizome of *Valeriana* was used in the experiment.

#### Reagent

Valtrate (wkq1805291), acevaltrate (wkq18020803), and homobaldrinal (wkq20083103) were obtained from Sichuan Weikeqi Biological Technology Co., Ltd. (Chengdu, China), and their purity was ≥98.0%. Baldrinal (215J9S63704) was obtained from Chengdu Must Bio-Technology Co., Ltd. (Chengdu, China), and its purity was ≥98.0%. 11-ethoxyviburtinal was prepared in our laboratory, and its purity was ≥98.0% (HPLC). Paroxetine hydrochloride was purchased from Huahai Pharmaceutical Co., Ltd. (Linhai, China). ELISA kits (Jiangsu Meimian Industrial Co., Ltd., China); TRIzol (Thermo Fisher, United States); PrimeScript™ RT reagent Kit (Takara, China); GoTaq^®^ Real-time PCR Systems (Promega Inc., United States) were also obtained.

#### Preparation of ZZX Extract

The dry plant material (200 g) was crushed, soaked in a 10-fold volume of 95% ethanol (1:10, w/v) for 30 min, and extracted with ultrasonic extraction for 30°C, 1°h, three times. The extract was filtered and then concentrated under the reduced pressure condition. The concentrated extract (ethanol-free) was freeze-dried, and the extraction rate was 10%. The ZZX extract was then stored at −20°C.

#### Chemical Analysis of ZZX Extract

The lyophilized powder of ZZX extract (0.5 g) was redissolved in 10 ml of methanol. Samples were then filtered with 0.22-µm nylon membrane filters and analyzed on an Agilent 1260 Infinity Ⅱ HPLC system (Agilent, United States). Separations were accomplished on an Agilent Zorbax SB-C18 column (4.6 mm × 250 mm × 5 µm) with a column temperature of 25°C and a flow rate of 1 ml/min. The mobile phase contained a mixture of acetonitrile (A) and ultrapure water with 0.1% formic acid (B). A gradient elution program was used (0–5 min, 70% B; 5–15 min, 45% B; 14–20 min, 45% B; 20–30 min, 25% B; 30–40 min, 20% B; 40–45 min, 20% B; 45–50 min, 5% B). The injection volume was 10 μl. The detector was set at 254 nm for the acquisition of chromatograms. The analysis of ZZX constituents was tested by HPLC using valtrate, acevaltrate, homobaldrinal, baldrinal, and 11-ethoxyviburtinal as standards. Peak areas of the samples were substituted into a standard curve, and the contents of valtrate, acevaltrate, homobaldrinal, baldrinal, and 11-ethoxyviburtinal in ZZX were determined to be 8.845, 13.361, 0.606, 0.500, and 0.489 mg/g, respectively. Further details of the five compounds are presented online (Supplementary Tables S3, S4, and Supplementary Figure S1).

#### Animals

Male C57BL/6J mice, weighing (20 ± 2) g, were obtained from SPF (Beijing) Biotechnology Co., Ltd. (License No. SCXK Beijing 2019-0010). All mice were maintained at an indoor temperature of 25°C, a humidity of 45–55%, and a circadian rhythm of 12 h.

### Animal Grouping and Administration Regimens

Ninety mice were acclimatized for a week and randomly divided into six groups, with 15 mice in each group: a control group, PTSD model group, ZZX low-dose (ZZX-L, 40 mg kg^−1^), ZZX medium-dose (ZZX-M, 80 mg kg^−1^), and ZZX high-dose group (ZZX-H, 120 mg kg^−1^), and paroxetine hydrochloride (PX) group (positive control). Paroxetine hydrochloride was dissolved in 0.9% (W/V) normal saline, and mice were given intragastric (IG) administration with a dose of 6 mg/kg/day and a volume of 20 ml kg^−1^ for 3 weeks (Han et al., 2015). ZZX extract was suspended in normal saline, and mice were given IG administration with different doses of 40, 80, and 120 mg/kg/day. ZZX was reported to exert significant antianxiety effects at the dose of 40–120 mg/kg (Zhai et al., 2016). The control group and the PTSD model group were given the same volume of normal saline. All experiments involving animals were performed in accordance with the National Institutes of Health’s Guide for the Care and Use of Laboratory Animals and were approved by the Animal Care and Use Committee of Beijing University of Chinese Medicine.

### SPS Combined With FS Procedure

Randomly selected mice were exposed to the SPS and FS procedure as previously described (Hawley et al., 2011) using severe and multimodal stressors to induce PTSD-like characteristics in mice. Animals were restrained for 2 h in 50-ml centrifuge tubes with a screw on top having numerous air holes. They were immediately forced to swim for 10 min in a transparent cylinder with a water temperature of about 25°C. After a recovery period of 15 min, the mice were exposed to anhydrous diethyl ether until loss of consciousness. After a recovery period of 15 min, except for the control group, mice in other groups were placed in context apparatus to adapt the environment for 3 min, exposed to 15 cycles of 10 s foot-shock at 1.0 mA, with a 10-s inter-shock interval, and then returned to their home cages after 2 min.

**FIGURE 2:**
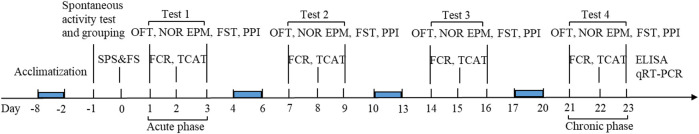
Schematic representation of the experimental procedure. OFT, open-field test; NOR, novel object recognition; FCR, fear-conditioning response; TCAT, traumatic cue avoidance test; EPM, elevated plus maze test; FST, forced swimming test; PPI, prepulse inhibition.

The mouse model of PTSD was established by the classical SPS and FS method. Based on the DSM-5 phenotypic model, including the six-dimensional anhedonia model (Liu et al., 2014), the six-dimensional externalized behavior model (Pietrzak et al., 2015), and the seven-dimensional hybrid model (Armour et al., 2015), seven behavioral tests of mice were observed in the experiment. The seven behavioral tests, including the open-field test (OFT), novel object recognition (NOR), fear-conditioning response (FCR), traumatic cue avoidance test (TCAT), elevated plus maze test (EPM), forced swimming test (FST), and pre-pulse inhibition (PPI), were used to investigate the states of mice, such as the anxiety, cognition, and memory, fear memory re-experience, avoiding cues, behavioral desperation, hypervigilance symptoms, and startle reflex, respectively. Mice with positive results on all seven tests developed PTSD. Behavioral tests were performed on days 1, 7, 14, and 21 after SPS and FS, and all seven symptoms were observed on day 21, with statistically significant differences among the groups. Finally, mice developed PTSD on day 21 after SPS and FS. Supplementary Methods, and results for the NOR, TCAT, and PPI were provided in the appendix. A schematic diagram of the experimental process is shown in Figure 2.

### Open-Field Test

The OFT is conducted to assess spontaneous locomotor activity and exploratory behavior in mice (Liu et al., 2021). The OFT apparatus consisted of a light-colored wooden box (50 × 50 cm) and a boundary wall (30 cm high). The bottom was divided into 25 equal squares by black lines. The mice were placed in the middle of the open field to explore arenas for 5 min, and the distance moved at the center and the total distance of movement of PTSD mice were recorded and analyzed using automated video tracking to evaluate exploratory behavior. The environment in the box was cleared after the end of the measurement.

### Elevated Plus Maze

The EPM has been well-validated to detect responses to anxiety and exploratory behavior in mice (Zhang Y. et al., 2021). A maze made entirely of stainless steel, painted blue, was positioned 40 cm above the ground and consisted of two open arms (30 × 5 cm each), two closed arms (30 × 5 cm each, arm height 25 cm), and a central platform (5 × 5 cm), arranged in the shape of a cross. For the EPM, a mouse was initially placed at the center, facing an open arm, and allowed to explore the arms for 5 min. The duration spent in each arm, and the total arm entries were consistently recorded.

### Forced Swim Test

The FST is described in previous reports (Li et al., 2020), and the reduction in immobility duration is identified with an antidepressant-like effect. Mice were forced to swim in a glass cylinder (diameter = 20 cm, water depth = 25 cm) filled with water 25 ± 1°C. Mice were not allowed to touch the bottom of the cylinder. Each mouse was forced to swim for 6 min, and the immobility time was recorded over the last 4 min.

### Fear Conditioning Response

Fear generalization is a symptom of anxiety-related disorders, including acute stress disorder and PTSD. This fear generalization was reflected by using the FCR (Yu et al., 2021). A fear conditioning apparatus consists of a stainless steel chamber (31 × 24 × 21 cm) with a stainless steel grid floor composed of bars (3.2 mm diameter) spaced 7.9 mm apart, allowing the delivery of electric foot-shocks. The front, back, and sides of the chamber were made of black acrylic. Except for the control group, mice in other groups were placed in context apparatus for 3 min during which they were exposed to electric shocks (3-s shock at 1 mA with a 33-s inter-shock interval for a total of five cycles) and noise. Two min later, they were sent back to mouse cages. 24 h after training, mice underwent a testing session, during which they were exposed to context apparatus (only the noise) for 5 min. The freezing time in the context apparatus was measured. The freezing time is defined as the absence of all movement, except breathing, and is used as a behavioral index of contextual fear conditioning (Yu et al., 2021).

### Hematoxylin–Eosin (HE) Staining

After the behavioral test was completed, the brains of four mice in each group were taken after heart perfusion. The brain tissues were fixed with paraformaldehyde, dehydrated with graded ethanol, embedded in paraffin, and then sliced continuously (3.3–4.5 mm anterior fontanelle) up to a thickness of 5 μm for HE staining. The morphology and structure of neurons in the hippocampal CA1 region were observed and photographed under a microscope.

## Network Pharmacology Analysis

### Screening of Chemical Ingredients of ZZX

The information of ingredients in ZZX was collected by TCMSP database (http://tcmspw.com/tcmsp.php) and BATMAN-TCM database (http://bionet.ncpsb.org.cn/batman-tcm/index.php/Home/Index/index). TCMSP is a systematic pharmacology platform designed for herbs (Ru et al., 2014). It provides multiple key absorption, distribution, metabolism, and excretion (ADME) properties such as drug-like properties, target, and related diseases of each compound. BATMAN-TCM database is an online tool for the study of molecular mechanisms of TCM. BATMAN-TCM compares submitted herbs to a list of reference ingredients saved in the database to predict targets for the new ingredients (Liu Z. et al., 2016). Active compounds with a cutoff score ≥20 and *p*-value ≤0.05 in BATMAN-TCM were selected. The pharmacokinetic and pharmacodynamic (ADME) properties of compounds were performed using QikProp 3.6 module from Schrodinger Maestro software. The potentially active ingredients were screened according to the Lipinski rules (MW < 500, H-bond donor <5, H-bond acceptor <10, logP (O/W) < 5, rotator <10) (Hsin et al., 2013). The final cluster of chemical ingredients of ZZX was a combination of BATMAN-TCM and TCMSP results.

### Target Prediction of the Active Ingredients of ZZX and PTSD

In this study, SwissTargetPrediction online analysis tool was used to predict the targets of potential active constituents in ZZX. SwissTargetPrediction (http://www.swisstargetprediction.ch/) is an online analysis platform for small-molecule target prediction, which can predict the targets of a large number of compounds based on the principle of molecular similarity (Channappanavar and Perlman, 2017)

The simplified molecular input specification (SMILES) information of 70 compounds was imported into the SwissTargetPrediction database to predict potential targets, and the macromolecules with probability >0.5 are taken as the potential targets of those components. GeneCards database (https://genecards.weizmann.ac.il/v3/), DrugBank database (https://www.drugbank.ca/), and TTD database (http://bidd.nus.edu.sg/group/cjttd/) were used to find targets related to PTSD. “Post-traumatic stress disorder” was used as the keyword to search and screen the PTSD-related targets. Known targets in the pathogenesis of PTSD were obtained. And then, all targets were submitted to the UniProt database (https://www.uniprot.org/) for the validation of their gene names.

### ProteinProtein Interaction Network Construction of Targets Associated With ZZX Activity on PTSD

The targets of ZZX on PTSD were submitted to STRING (https://string-db.org/cgi/input.pl). STRING is a database of known and predicted PPIs, including both direct and indirect interactions among proteins (Szklarczyk et al., 2017). The targets were input the STRING database, the organism was set to “Homo sapiens,” and a confidence score of 0.4–0.7 was set. Then, the PPI results exported as a TSV (.tsv) file were imported into Cytoscape (version 3.8.0) for further analysis, and the plugin “clustermaker” was applied to the layout of PPI network.

### Interaction Network Construction of ZZX Active Compounds Target for PTSD

Network analysis is conducive to scientific interpretation of the complex relationships among herbs, compounds, diseases, genes, and pathways (Li et al., 2014). Cytoscape 3.8.0 software (http://www.cytoscape.org/) is an open bioinformatics analysis software for visualizing molecular interaction networks, and it provides a set of basic data integration, analysis, and visualization functions to analyze complex networks. The two major parameters “degree” and “edge” are conducted to evaluate its topological features for each node in the interaction network. A degree is defined as the number of edges to node i (Liu H. et al., 2016). The higher the degree is, the more important the component or target is in the network.

### Gene Ontology and Kyoto Encyclopedia Genes Genomes Enrichment and Network Construction of Active Compounds–Targets–Pathway Associated With ZZX Activity on PTSD

To illustrate the role of potential targets in gene function and signal pathways, the related targets were imported into DAVID (https://david.ncifcrf.gov/home.jsp) database for enrichment analysis of Gene Ontology (GO) and Kyoto Encyclopedia of Genes and Genomes (KEGG). Gene names were corrected to official gene names by inputting the list of target gene names and defining the species as “Homo sapiens.” After the aforementioned database retrieval and transformation operation, the threshold was set as *p* < 0.05. Finally, R software was used to visualize the analysis results by drawing a bubble chart. The “component–target–pathway” network was constructed by using Cytoscape 3.8.0 software to further analyze the relationship among the key components, targets, and pathways of ZZX acting on PTSD.

## Experimental Verification

### Quantification of Neurotransmitter and HPA in Different Brain Regions and Serum by ELISA

After the last behavioral experiment, mice were killed with decapitation. Blood was collected by decapitating mice, and the serum was separated by centrifugation. The hippocampus of each mouse was isolated and frozen separately in liquid nitrogen. The levels of 5-hydroxytryptamine (5-HT), dopamine (DA), norepinephrine (NE), γ-aminobutyric acid (GABA), and glutamic acid (Glu) in the brain of mice and the levels of adrenocorticotropic hormone (ACTH), corticosterone (CORT), and corticotropin-releasing hormone (CRH) in the brain and serum were measured using ELISA kits. The operation was strictly in accordance with the corresponding instructions, and the concentration was normalized according to the standard curve.

### Detection of *CB1*, *DAGLα*, *MAGL*, and *FAAH* mRNA in Hippocampus by Real-Time PCR

Total RNA was extracted by TRIzol reagent from the hippocampus of mice. The concentrations and purity of RNA were determined, and the quality of RNA was evaluated by the ratio of absorbance at 260–280 nm and ranged from 1.9 to 2.1. Total RNA samples were reverse-transcribed, and the cDNA was obtained using a PrimeScriptTM RT reagent Kit. The real-time PCR reaction was conducted according to the GoTaq^®^ Real-time PCR Systems Kit instructions. The expression levels of *CB1*, *DAGL-α*, *MAGL*, and *FAAH* mRNA in the hippocampus were determined on real-time PCR system (Bio-Rad icycler IQ, United States). The sequences of the primers used for real-time PCR are shown in Table 1. Transcript levels of target genes were quantified by using the 2^−ΔΔCt^ value method.

**TABLE 1:** Primer information for the real-time PCR experiment.

Gene name	Forward primer sequence	Reverse primer sequence
CB1	5′-CTG​AGG​GTT​CCC​TCC​CGG​CA-3′	5′-TGC​TGG​GAC​CAA​CGG​GGA​GT-3′
FAAH	5′-GCC​CTT​CAG​AGA​GCA​GCT​CT-3′	5′-CTT​TTC​AGC​TGA​CCG​AGG​AC-3′
DAGLα	5′-CAC​GAG​ATG​CTA​CGC​TAC​AAA​GA-3′	5′-GGC​AGA​GAC​AAC​ACG​AGC​A-3′
MAGL	5′-CGG​AAC​AAG​TCG​GAG​GTT​GA-3′	5′-TGT​CCT​GAC​TCG​GGG​ATG​AT-3′
*β*-actin	5′-ATG​GTG​GGT​ATG​GGT​CAG​AA-3′	5′-TCC​ATA​TCG​TCC​CAG​TTG​GT-3′

### Statistical Analysis

Data were analyzed, and plots were generated using GraphPad Prism version 6.0, and all summary data are presented as mean ± SEM from 10 to 15 independent experiments. Required sample size of each experiment was estimated by power calculations based on data from previous studies and preliminary experiments with an alpha level of 0.05 and 80% power. Normality of data distribution was examined using the Shapiro–Wilk normality test. Normally distributed data were analyzed using the unpaired, two-tailed Student’s t-test (for comparing two groups), or one-way ANOVA with Bonferroni post hoc test (for multigroup comparisons). For data not following a normal distribution, the unpaired, two-tailed Mann–Whitney *U* test (for comparing two groups), or Kruskal–Wallis test (for multi-group comparisons) was used. Representative images were selected based on the value closest to the mean value of each group. *p* values are reported in the figures, and a value <0.05 was considered statistically significant.

## Results

In this study, we used SPS and FS to establish a mouse model of PTSD. Moreover, we also investigated the anti-PTSD pharmacological action of ZZX by behavioral experiments and histopathological examinations of hippocampus.

### Effects of ZZX on the Movement Distance and Numbers of Upright in the OFT

**FIGURE 3:**
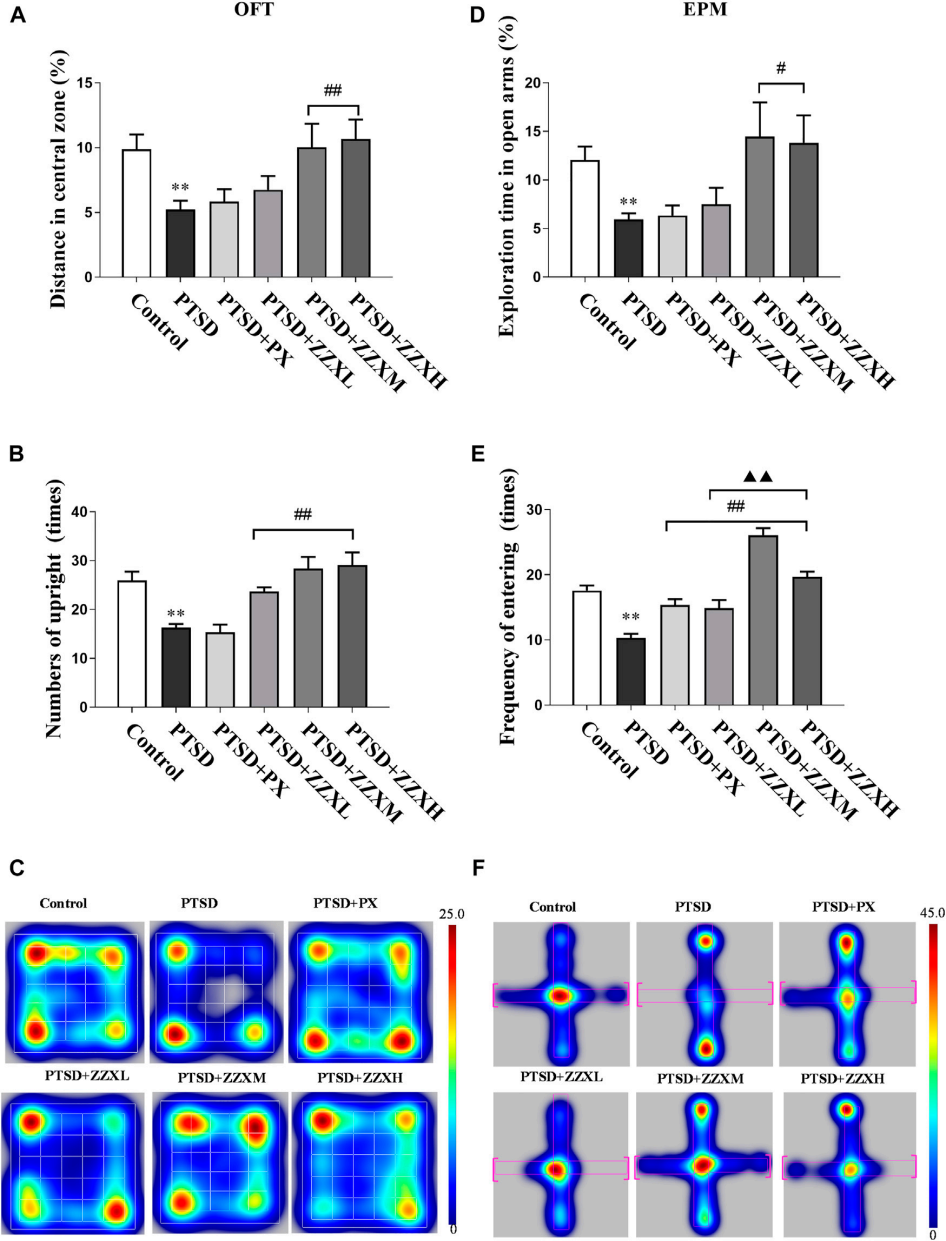
Effects of SPS and FS and ZZX treatment (40, 80, 120 mg kg^−1^) on OFT and EPM. **(A)** Distance in central zone; **(B)** numbers of upright; **(C)** heat map of the trajectory of an individual mouse over a 5-min testing session in the OFT; **(D)** exploration time in open arms; **(E)** frequency of entering; **(F)** heat map of the trajectory of an individual mouse during the EPM. The horizontals refer to open arms, and the verticals refer to closed arms. Values are expressed as (mean ± SEM, *n* = 10–15). *p < 0.05, **p < 0.01 vs control group. ^#^ *p* < 0.05, ^##^ *p* < 0.01 vs PTSD group. ▲*p* < 0.05, ▲▲*p* < 0.01 vs ZZXM group.

Analysis of the OFT revealed a significant decrease in both movement distances in the center and the standing upright of PTSD mice compared to the control group (*p* < 0.01) (Figures 3A–C). In contrast, we observed a significant increase in the movement distance and numbers of standing upright in the ZZX-M and ZZX-H treatment (*p* < 0.01), as compared to the PTSD group, while the PX group was less effective than ZZX-M and ZZX-H (Figures 3A,B).

### Effects of ZZX on the Exploration Time and Frequency of Entering in the EPM

Compared with the control group, the PTSD group had a significant reduction in the distance moved (Figure 3F), cumulative duration, and frequency of entering the open arm (*p* < 0.01) (Figures 3D,E). ZZX-M and ZZX-H treatment significantly increased the cumulative duration on the open arm, as compared to the PTSD group (*p* < 0.05), while the PX group was less effective than ZZX-M and ZZX-H (Figure 3D). In addition, all doses of ZZX significantly increased the frequency of entering the open arm (*p* < 0.01), but the PX group was less effective than ZZX-M and ZZX-H (Figure 3E).

### Effects of ZZX on the Immobility Time in the FST

**FIGURE 4:**
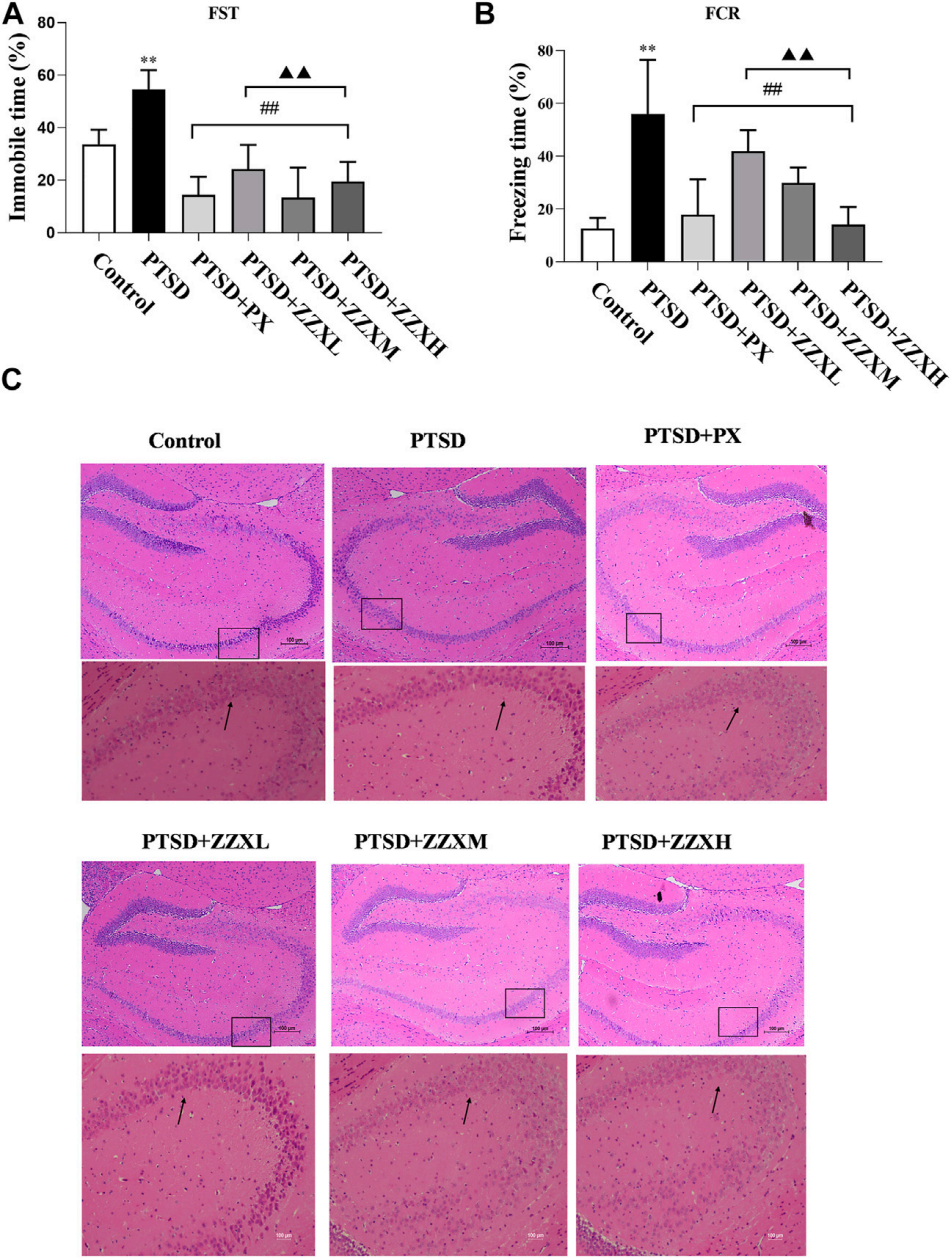
Effect of ZZX on FST, FCR, and pathological changes in hippocampus CA1 region of mice. **(A)** Immobile time in the FST; **(B)** freezing time in the FCR; **(C)** hematoxylin–eosin (HE) staining (magnification: ×200, above; magnification: ×400, below). Values are expressed as (mean ± SEM, *n* = 10–15). *p < 0.05, **p < 0.01 vs control group. ^#^ *p* < 0.05, ^##^ *p* < 0.01 vs PTSD group. ▲*p* < 0.05, ▲▲*p* < 0.01 vs ZZXM group.

The FST revealed a significant increase in the immobility time of PTSD mice, as compared to the control group (*p* < 0.01) (Figure 4A). Furthermore, the immobility time was significantly decreased in both doses of ZZX and PX treatments as compared to PTSD mice (*p* < 0.01) (Figure 4A). There were significant differences among different doses of ZZX (*p* < 0.01), and the immobility time of mice in the ZZX-M group was the lowest, followed by ZZX-H and ZZX-L (*p* < 0.01) (Figure 4A). The efficacies of the ZZX-M and PX treatments were similar (Figure 4A).

### Effects of ZZX on the Freezing Time in the FCR

For the FCR test, PTSD mice showed significantly increased freezing time, as compared to the control group (*p* < 0.01) (Figure 4B). Compared with the PTSD group, mice in different doses of ZZX and PX treatments had a significant reduction in the freezing time (*p* < 0.01) (Figure 4B). Furthermore, there were significant differences between different doses of ZZX, and the freezing time of the ZZX-H group was the lowest, followed by ZZX-M and ZZX-L (*p* < 0.01) (Figure 4B). The PX group was less effective than the ZZX-H treatment (Figure 4B).

### Effects of ZZX on Histopathological Characteristics of Hippocampal CA1 Region in Mice

HE staining showed that neurons in the hippocampal CA1 region of control mice were closely arranged with a clear intracellular structure (Figure 4C; control). However, neurons in the model group were irregular and pyknotic and revealed a disordered arrangement and an incomplete structure (Figure 4C PTSD). After administrating ZZX and PX, the neuronal morphology was improved with a complete structure as compared to the model group (Figure 4C PTSD+PX, ZZXL, ZZXM, ZZXH). Moreover, the higher the concentration of ZZX administration, the more effective it was. The effect of PX was similar to that of the ZZX-M group (Figure 4C PTSD+PX, ZZXM).

### Network Pharmacology Assay

Furthermore, network pharmacology analysis was used to predict the potential active ingredients, targets, and possible pathways of ZZX in the treatment of PTSD.

#### Active Chemical Ingredients of ZZX

A total of 70 active ingredients were obtained by screening under the conditions of ADME properties and the Lipinski rules, and the information of 19 components with more edge connections in the ZZX active compounds-target proteins network is listed in Table 2.

**TABLE 2:** Information of the main active ingredients in *Valeriana jatamansi* Jones ex Roxb.

No.	PubChem CID	Name	Rotor	QPlogPo/w	accptHB	Donor HB	Mol MW
1	159846	Baldrinal	3	1.169	4.50	0	218.209
2	13455460	Chlorovaltrate K	9	3.811	8.45	1	460.951
3	65689	Didrovaltrate	8	2.808	9.70	0	424.490
4	5281642	6-hydroxyluteolin	5	0.313	5.25	4	302.240
5	5280863	Kaempferol	4	1.053	4.50	3	286.240
6	49999	Homobaldrinal	5	2.438	4.50	0	260.289
7	3084294	Homodidrovaltrate	9	3.360	9.70	0	438.517
8	12299954	*S*-fukinolide	4	2.956	7.50	0	408.509
9	38356815	Glycosmisic acid	8	2.653	6.70	3	372.374
10	442436	Valtrate	8	2.986	9.70	0	422.474
11	5280443	Apigenin	3	1.652	3.75	2	270.241
12	5280442	Acacetin	3	2.508	3.75	1	284.268
13	5281612	Diosmetin	4	1.812	4.50	2	300.267
14	5280445	Luteolin	4	0.928	4.50	3	286.240
15	3010930	8-hydroxy-pinoresinol	5	2.514	7.15	3	374.390
16	122169319	Decursitin D	3	2.509	6.95	1	344.363
17	6440940	Valerenic acid	2	3.623	2.00	1	234.338
18	65717	Acevaltrate	9	2.120	11.70	0	480.511
19	445858	Ferulic acid	5	1.374	3.50	2	194.187

#### Analysis of Protein–Protein Interaction Network and Potential Targets of ZZX on PTSD

**FIGURE 5:**
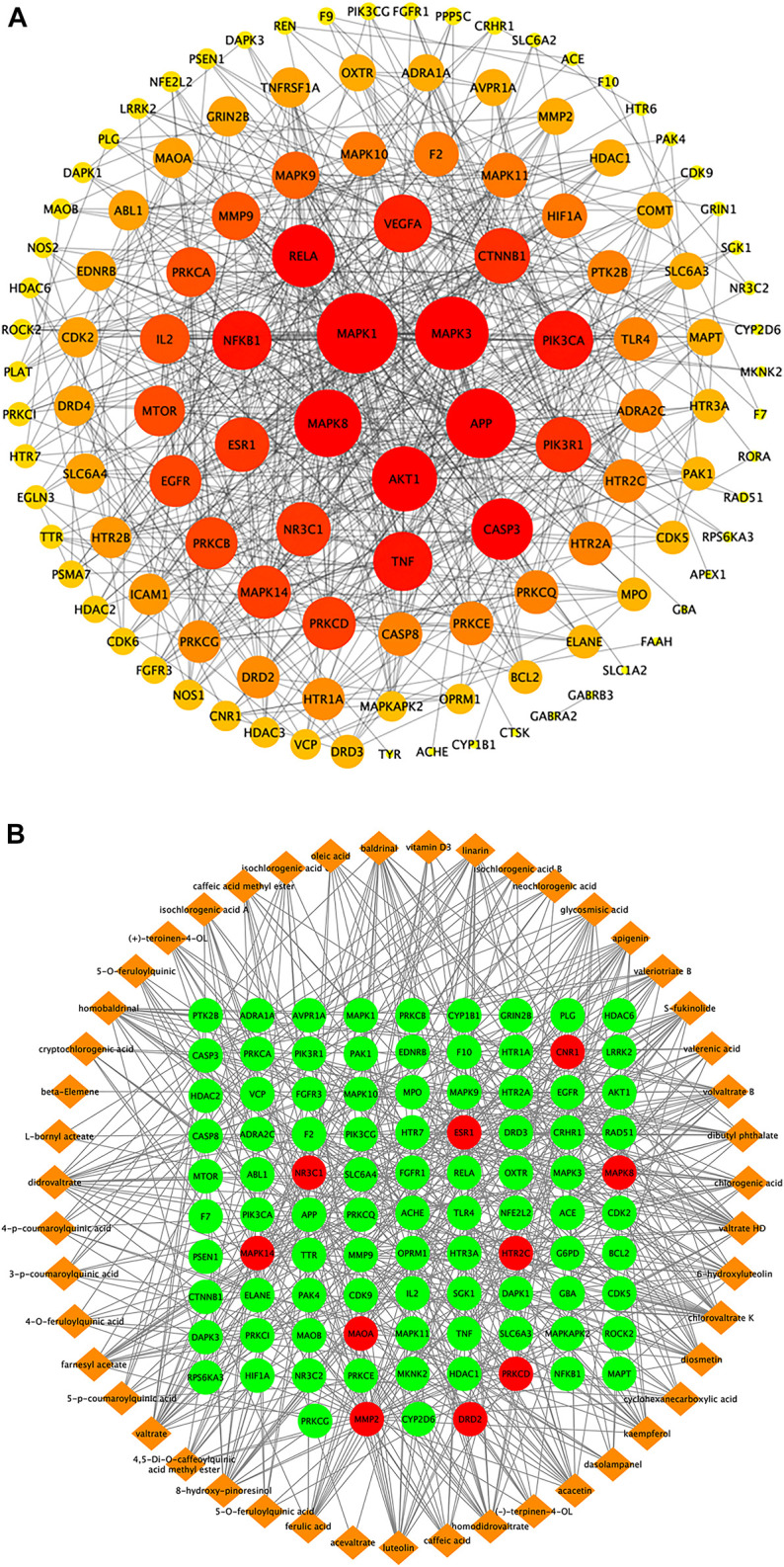
Target proteins interaction network, ingredient-target network, and compound–target–pathway network of potential targets for primary active ingredients from ZZX against PTSD. **(A)** Interaction network of ZZX target proteins involved in PTSD; **(B)** ZZX active ingredient–target network associated with PTSD.

The area total of 662 promising targets of ZZX was retrieved from TCMSP, SwissTargetPrediction, and BATMAN-TCM. The area total of 311 targets related to PTSD was retrieved from DrugBank, TTD, and GeneCards database. To explore the relationship between ZZX and PTSD, the area total of 130 common targets was identified. The network of ZZX acting on PTSD consisted of 120 nodes and 659 edges. The larger the node, the more important it is (Figure 5A). The larger nodes such as MAPK1 (mitogen-activated protein kinase 1), nuclear receptor subfamily 3 group C member 1 (NR3C1), 5-hydroxytryptamine receptor 2A (HTR2A), 5-hydroxytryptamine receptor 2C (HTR2C), and estrogen receptor alpha (ESR1) indicated that they played an important role in anti-PTSD activity.

#### Network Analysis of ZZX Active Compound–Target Proteins Implicated in PTSD

The “ZZX active compounds–target proteins” network consisted of 141 nodes and 568 edges with 47 active compounds presented in orange and 94 targets in green (Figure 5B). The more common target proteins were solute carrier family six member 4 (SLC6A4), CNR1, monoamine oxidase A (MAOA), NR3C1, mitogen-activated protein kinase 14 (MAPK14), c-Jun N-terminal kinase 1 (MAPK8), HTR2A, HTR2C, D2 dopamine receptor (DRD2), matrix metalloproteinase 2 (MMP2), δ protein kinase C delta (PRKCD), and ESR1. The more common active compounds were baldrinal, homobaldrinal, didrovaltrate, valtrate, chlorovaltrate K, 6-hydroxyluteolin, apigenin, acacetin, diosmetin, luteolin, kaempferol, 8-hydroxypinoresinol, and glycosmisic acid.

#### Gene Ontology Enrichment and Kyoto Encyclopedia Genes Genomes Pathway Analysis of Targets of ZZX against PTSD

**FIGURE 6:**
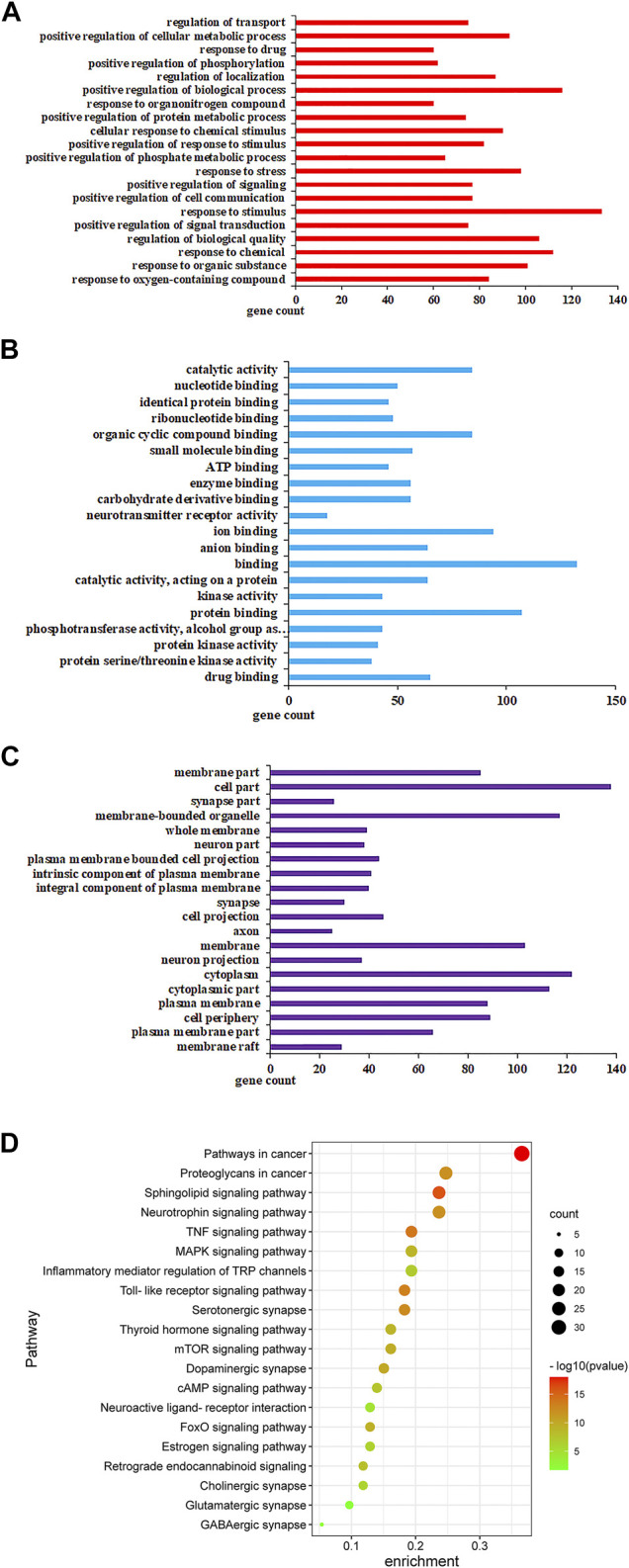
GO analysis and KEGG analysis of potential targets for primary active ingredients from ZZX against PTSD. **(A)** Biological processes (BP); **(B)** molecular function (MF); **(C)** cellular components (CC); **(D)** KEGG enrichment analysis.

The 94 targets were analyzed on three levels: biological processes (BP), molecular function (MF), and cellular components (CC). Pathways identified under the BP category included response to stimulus, response to chemical, positive regulation of the biosynthetic process, and cellular metabolic process (Figure 6A). Pathways identified under the MF category included catalytic activity, ion binding, protein binding, organic cyclic compound binding (Figure 6B). Pathways identified by the CC category included membrane-bounded organelle, cytoplasm, plasma membrane (Figure 6C). Pathways that showed *p* < 0.05 were selected as potential pathways implicated in the activity of ZZX against PTSD. These pathways are implicated in serotonergic synapse, glutamatergic synapse, dopaminergic synapse, serotonergic synapse, and retrograde endocannabinoid signaling (Figure 6D).

### Effects of ZZX on the Levels of 5-HT, NE, DA, GABA, and Glu

**FIGURE 7:**
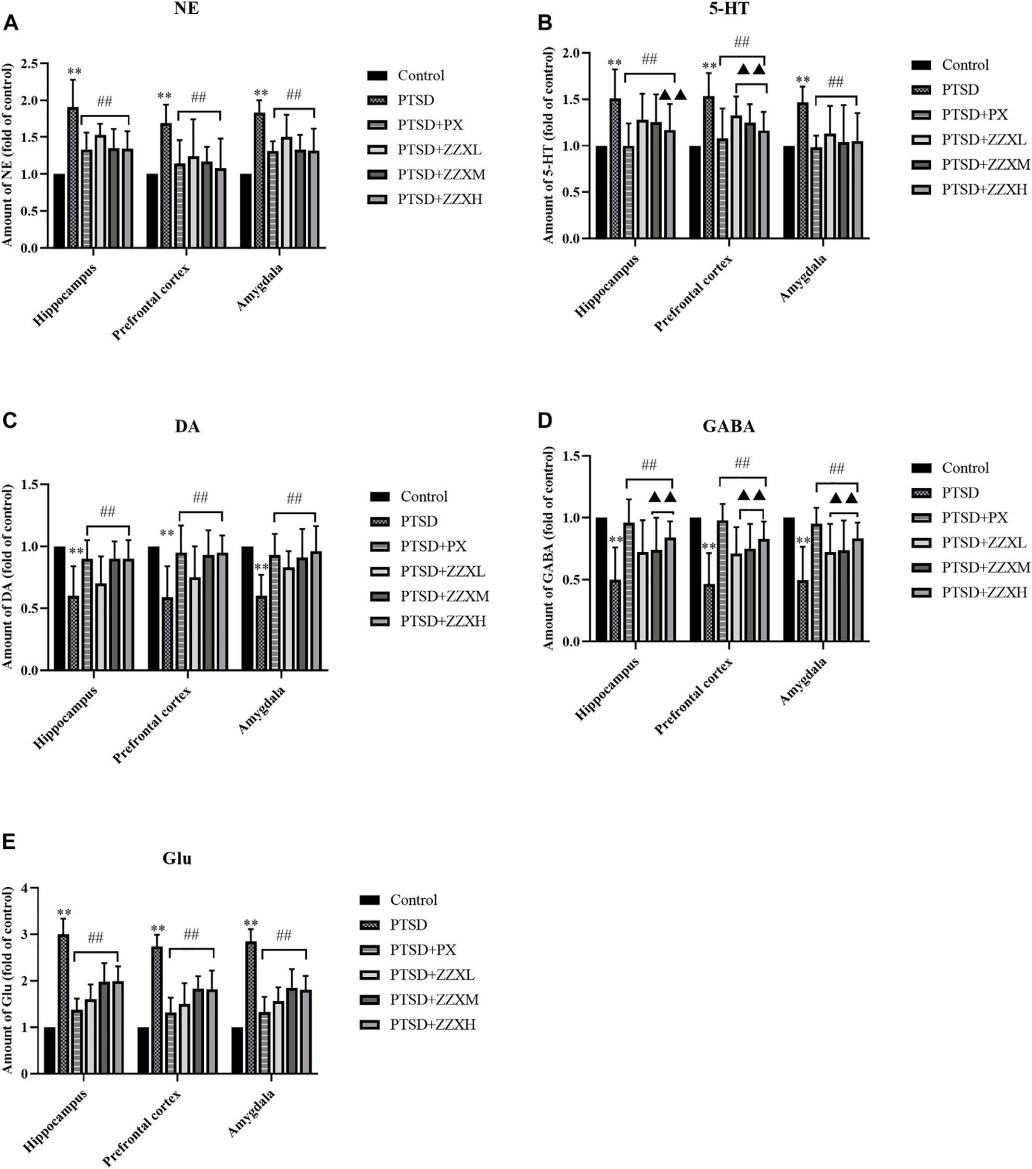
Effects of ZZX treatment on 5-HT, NE, DA, GABA, and Glu **(A**–**E)** levels in different brain regions of mice. Values are expressed as (mean ± SEM, *n* = 10–15). *p < 0.05, **p < 0.01 vs control group. ^#^ *p* < 0.05, ^##^ *p* < 0.01 vs PTSD group. ▲*p* < 0.05, ▲▲*p* < 0.01 vs ZZXM group.

Network pharmacology was used to predict the active mechanisms of ZZX against PTSD by constructing a complex network relationship featuring ZZX, active ingredients, targets, and PTSD. However, this was only a virtual screening result. Due to the lack of falsification mechanisms in network pharmacology, *in vivo* tests were also performed in mice. The expression levels of neurotransmitters, hormones, and key target genes were detected by ELISA and real-time PCR to further verify this inference. Analysis of ELISA showed that NE, 5-HT, and Glu levels of PTSD mice had a significant increase in the hippocampus, prefrontal cortex, and amygdala, as compared to normal mice (*p* < 0.01) (Figures 7A,B,E), while levels of DA and GABA were significantly decreased (*p* < 0.01) (Figures 7C,D). Furthermore, brain levels of 5-HT, NE, DA, GABA, and Glu of PTSD mice were significantly reversed by PX and all doses of ZZX (*p* < 0.01) (Figures 7A–E). PX and ZZX-H had better effects on 5-HT and GABA (*p* < 0.01) (Figures 7B,D).

### Effects of ZZX on the Levels of ACTH, CORT, and CRH

**FIGURE 8:**
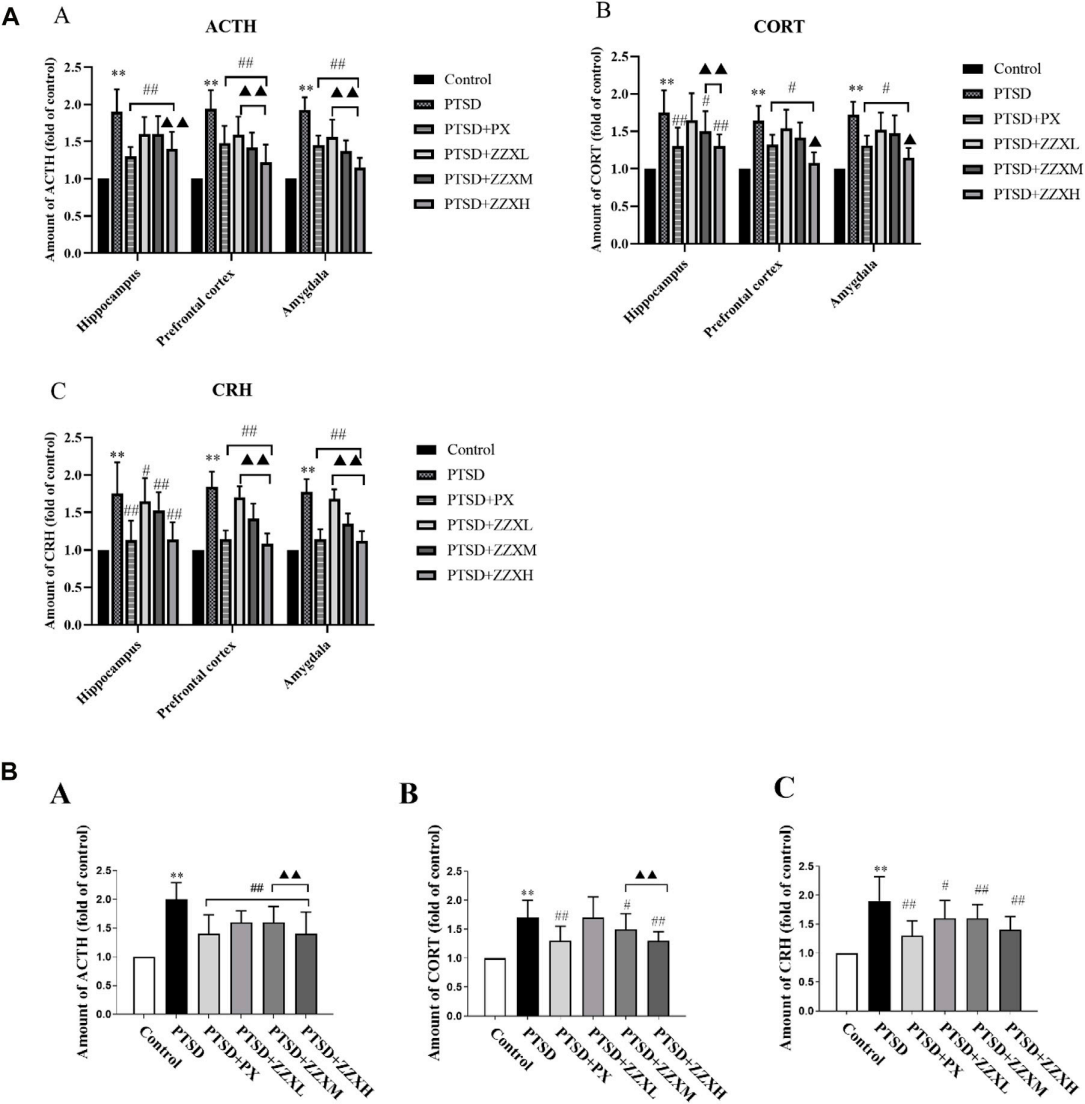
**(A)** Effects of ZZX treatment on ACTH, CORT, and CRH (A, B, C) levels in different brain regions of mice. Values are expressed as (mean ± SEM, *n* = 10–15). *p < 0.05, **p < 0.01 vs control group. ^#^ *p* < 0.05, ^##^ *p* < 0.01 vs PTSD group. ▲*p* < 0.05, ▲▲*p* < 0.01 vs ZZXM group. **(B)** Effects of ZZX treatment on ACTH, CORT, and CRH (A, B, C) levels in serum of mice. Values are expressed as (mean ± SEM, *n* = 10–15). *p < 0.05, **p < 0.01 vs control group. ^#^ *p* < 0.05, ^##^ *p* < 0.01 vs PTSD group. ▲*p* < 0.05, ▲▲*p* < 0.01 vs ZZXM group.

Levels of ACTH, CORT, and CRH significantly increased in the hippocampus, prefrontal cortex, and amygdala of PTSD mice, as compared to the control group (*p* < 0.01) (Figure 8A A∼C). Nevertheless, levels of ACTH, CORT, and CRH were significantly decreased in PX and ZZX treatments; the efficacies of ZZX-H and PX treatments were similar, followed by ZZX-M and ZZX-L (*p* < 0.01) (Figure 8A A∼C).

For hormone levels in serum of mice, compared with the PTSD group, mice in different doses of ZZX and PX treatments had a significant reduction in the levels of ACTH, CORT, and CRH (*p* < 0.01) (Figure 8B A∼C). Furthermore, for ACTH and CORT, the efficacies of the ZZX-H and PX groups were similar (*p* < 0.01) (Figure 8B A, B).

### Effects of ZZX on the Expression of *CB1*, *DAGLα*, *MAGL*, and *FAAH* mRNA in the Hippocampus of Mice

**FIGURE 9:**
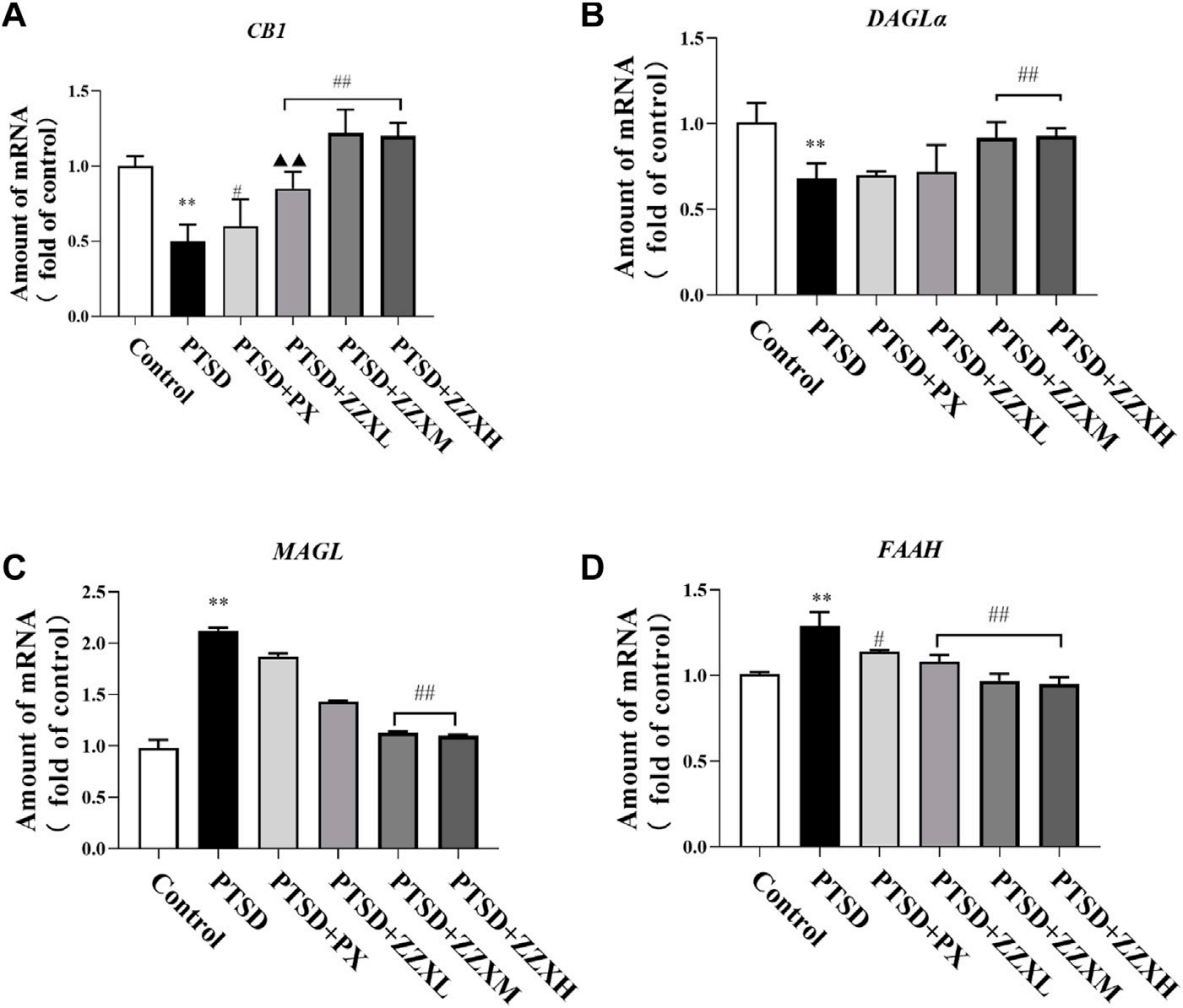
Effects of ZZX treatment on CB1 **(A)**, DAGLα **(B)**, MAGL **(C)**, and FAAH **(D)** mRNA expression. Values are expressed as (mean ± SEM, *n* = 5). ^*^ *p* < 0.05, ^**^ *p* < 0.01 vs control group. ^#^ *p* < 0.05, ^##^ *p* < 0.01 vs PTSD group. ▲*p* < 0.05, ▲▲*p* < 0.01 vs ZZXM group.

Analysis of real-time PCR revealed a significant decrease in the levels of *CB1* and *DAGLα* mRNA in the PTSD group (*p* < 0.01), as compared to the control group, while the levels of *FAAH* and *MAGL* mRNA were significantly increased (*p* < 0.01) (Figures 9A–D). In contrast, expression levels of *CB1* and *DAGLα* mRNA in the ZZX-M and ZZX-H group and the level of *CB1* mRNA in the PX group were significantly increased compared with the PTSD group (*p* < 0.01, *p* < 0.05) (Figures 9A,B), while expression levels of *FAAH* and *MAGL* mRNA in the ZZX-M and ZZX-H groups and the level of *FAAH* mRNA in the PX group were significantly decreased (*p* < 0.01, *p* < 0.05) (Figures 9C,D).

## Discussion

### Evaluation of Animal Models of PTSD

In this study, SPS combined with FS was used to prepare a mouse model of PTSD. The OFT, EPM, FST, and FCR were used to evaluate whether the PTSD model was successful. Experimental results showed that SPS combined with FS effectively stimulated the process of traumatic events and induced the main symptoms of PTSD, including negative emotions, avoiding cues that could indicate trauma, strong vigilance, loss of interest, startle, etc. These symptoms completely appeared on the 21st day, and in contrast to the normal control group, mice with all of these significant symptoms were diagnosed with PTSD. Current medication for PTSD is suboptimal in terms of efficiency and tolerability (Sartori and Singewald, 2019), highlighting the need for improved drug treatments.

### Antagonistic Effects of 95% Ethanol Extract of ZZX on PTSD-like Behavior in Mice

Traditional Chinese medicine studied in different phases of clinical trials for their potential in the treatment of fear-, anxiety-, and trauma-related disorders is adopted. Furthermore, several experimental studies suggest that ZZX (Jugran et al., 2019) and its active ingredient valtrate (Zhang et al., 2018) play a role in several forms of nervous system diseases, including negative emotions related to anxiety and depression. Clinic-based studies suggest that patients with depression benefit from 70% ethanol extract of ZZX (Bhattacharyya et al., 2007). Due to PTSD accompanied by negative emotions and anhedonia, we found that PTSD was prevented by treating the mice with a 95% ethanol extract of ZZX. Thus, the reduced activity, time for exploration, and movement distance in PTSD mice could explain the clinically relevant changes in PTSD-related phenotype. Moreover, ZZX therapy in PTSD mice restored the movement distance in the OFT, time for exploring in the EPM, and immobility time in the FST and FCR, significantly reducing negative emotions, strong vigilance, and loss of interest. Here, using HE staining, we demonstrate that administering ZZX in PTSD mice partially restores the neural cell in the hippocampus. Although ZZX therapy in the PTSD has long been overlooked, our results suggest that ZZX may serve as a novel therapeutic drug for PTSD.

### Analysis of Main Active Ingredients

One strategy followed in drug development is refining and improving compounds interacting with existing anti-PTSD drug targets, such as serotonergic. A more innovative approach involves the search for compounds with novel mechanisms of anti-PTSD action using the growing compound library and disease databases concerning the relevant neurocircuitries and neurobiological mechanisms underlying pathological fear and anxiety. Although contents of five main ingredients in ZZX have been detected, we also found that 47 active ingredients from network pharmacology in the treatment of PTSD were mainly iridoids, flavonoids, and chlorogenic acids. Previous studies also revealed that valtrate, isochlorogenic acid A, and hesperetin play a role in anxiolytic action mediated by corticosterone levels (Shi et al., 2014; Wang et al., 2018); here, we found that the hormone levels on the HPA axis are significantly increased in PTSD mice, which suggests that the HPA axis may also be important in PTSD. Importantly, acevaltrate has been shown to scavenge oxygen-free radicals, reduce oxidative stress, and play a neuroprotective role in the antioxidant activity mechanism (Wang et al., 2017). In addition, acacetin was found to increase protection to mitochondria-mediated apoptosis in nerve cells induced by 6-hydroxydopamine (Kim et al., 2017). Thus, our finding that ZZX protects PTSD mice from negative emotions and hippocampus injury suggests that PTSD may be targeted using a combination of valtrate, baldrinal, and phenolic acid therapy.

### Main Targets and Pathways Analysis

The target systems evaluated in network pharmacology include glutamate, eCB, and neuropeptide systems, as well as ion channels and targets derived from phytochemicals. As the critical protein of the ECS, CB1 is mainly expressed in the GABAergic and glutamatergic systems of the hippocampus and amygdala. Based on previous studies, loss of eCB in the brain can lead to PTSD. For example, eCB is downregulated in numerous models of fear-, depression-, anxiety-, and trauma-related disorders, and inhibiting the synthesis of eCB by overexpressing FAAH significantly increases sensitivity to negative emotions, contextual fear memory, loss of interest, etc. (Korem and Akirav, 2014; Segev et al., 2014; McEwen et al., 2015). Importantly, activating the ECS by overexpressing CB1 has been shown to alleviate the HPA axis and neural circuit damage in several clinical models, including models of acute and chronic stress (Jenniches and Zimme, 2016; Balsevich et al., 2017). These previous results also provide insights into the pathogenic mechanism that underlies loss of eCB in PTSD, showing that both overexpressing CB1 and treatment with FAAH inhibitors can protect against PTSD. In addition, the link between cholinergic synaptic signaling and regulation of glutamate and GABA neurons remains an open question in the increased risk of developing Alzheimer’s disease (Herrup, 2015).

Serotonin (5-HT) synaptic pathway is composed of serotonin and various serotonin receptors, which mediate higher brain activities such as memory, learning, behavior, emotion, and its functional changes often lead to diseases such as depression, anxiety, and Alzheimer’s disease (Kohler et al., 2016). The activation of 5-HT2A receptor releases 5-HT and induces the HPA axis causing anxiety effect (Adamec et al., 2004). Furthermore, as a 5-HT transporter, SLC6A4 is a critical target of the major depressive episode (Consoloni et al., 2018), and this protein may serve as a novel therapeutic target for PTSD (Lam et al., 2018). Similarly, DRD2 is involved with anxiety, depression, and social dysfunction (Lawford et al., 2006). It is noteworthy that MAOA and MAOB are pharmacological targets of monoamine oxidase inhibitors for clinical antidepressants and play a key role in the metabolism of monoamine neurotransmitters (Ziegler and Domschke, 2018). Interestingly, ESR is the main mediator of estrogen receptors (Paterni et al., 2014) and inhibits the damage of excessive glutamate through the estrogen-signaling pathway. Especially, ESR subtypes, ERα and Erβ, play a central role in regulating brain-derived neurotrophic factors and 5-HT signaling pathway (Chhibber et al., 2017).

Indeed, examples of promising novel candidates currently in clinical development for generalized anxiety disorder, social anxiety disorder, panic disorder, obsessive-compulsive disorder, or post-traumatic stress disorder include ketamine, riluzole with one common pharmacological action of modulation of glutamatergic neurotransmission. Finally, compounds such as D-cycloserine, cannabinoids have shown efficacy in enhancing fear-extinction learning in humans (Sartori and Singewald, 2019).

In the study of network pharmacology, cell dysfunction, metabolic dysfunction, and neuronal stem cell have been linked to nervous system disease, and inflammation has also been postulated to be among the factors connecting neural stem cell dysfunction to PTSD. Nevertheless, the underlying mechanisms remain unclear. Although network pharmacology plays a role in clinical trials of new drugs, given the fact that there are as yet no means to verify these hypotheses, these candidates, targets, and action pathways remain highly speculative.

### Effects of 95% Ethanol Extract of ZZX on Neurotransmitters and HPA Axis

Using SPS and FS as a mouse model of PTSD, we showed that SPS and FS lead to paucity in DA of the cerebral limbic system, but the treatment of ZZX restores DA levels. Moreover, several previous studies have uncovered that increased levels of DA release cause reward-oriented or aversion-oriented motivational behavior (Parsons and Hurd, 2015). Additionally, a finding of our study is that mice induced by SPS and FS developed an altered neurotransmitter homeostasis characterized by excessive Glu. Furthermore, previous studies have reported that excessive Glu leading to excitotoxicity worsens brain damage and contributes to long-term neurological deficits (Sun et al., 2021). Importantly, our results suggest that ZZX improves long-term neurologic deficits through antagonizing glutamate-induced excitotoxicity. It has been reported that forgetting of fear memory is a current medical therapy for PTSD, and both hippocampal long-term depression and GABAergic transmission may be the underlying mechanism (Cao et al., 2021). Specifically, ZZX modulates fear memory forgetting by increasing the brain level of GABA in the context of PTSD. Consistent with these results, we found that DA and GABA in the brain of mice treated with ZZX were increased; however, 5-HT, Glu, and NE were significantly reduced. Our data demonstrated a link between neurotransmitters, and PTSD-like behavior provides critical support for the potential feasibility of administering ZZX to treat PTSD.

In further studies, we found that ACTH, CORT, and CRH in serum and specifically in the brain were increased during PTSD, which suggests that they may exert negative effects on mice. Recently, chronic corticosterone administration was shown to contribute to anxiety and depression and was sufficient to impair adult neurogenesis (Siopi et al., 2016). Additionally, as a regulator of the HPA has been previously shown to prevent depression and anxiety disorders in the face of acute and chronic stress (Shi et al., 2014), we examined the effect of ZZX on this response. As expected, ZZX led to protection in the face of PTSD. These data suggest that PTSD-like phenotypes driven by neurotransmitters and the HPA may be a consequence of injury occurring locally in specific regions of the brain.

### Effects of 95% Ethanol Extract of ZZX on Endocannabinoid-Related Genes

Here, we show that the SPS and FS are sufficient to initiate a pathological feed forward loop for PTSD by impairing the ECS in the hippocampus, a brain region strongly involved in the development of depressive symptoms. In the hippocampus, we observed a specific decrease in *CB1* and *DAGLα* gene—but an increase in *FAAH* and *MAGL* gene—in PTSD mice induced by SPS and FS. Importantly, the ECS has been reported to regulate mood, emotions, and responses to stress through the activation of the cannabinoid receptor CB1. For instance, the CB1 receptor antagonist rimonabant, initially prescribed for the treatment of obesity and associated metabolic disorders, increases the incidence of depressive symptoms. Furthermore, treatment before stress sessions with rimonabant prolonged freezing responses of stressed mice during cued fear recall tests (Dubreucq et al., 2012). In contrast, low doses of synthetic CB1 agonists—but not high doses—produce anxiolytic- and antidepressant-like effects in animal models (Chevalier et al., 2020). In particular, chronic stress has been shown to decrease eCB signaling in the brain. Altogether, our result is consistent with pharmacological observations reporting low brain levels of *CB1* gene in rodents suffering from depression, post-traumatic stress disorder, or chronic stress. Moreover, our data imply that it is the therapeutic properties of ZZX that improves *CB1* and *DAGLα* gene, decreases *MAGL* and *FAAH* gene, and alleviates PTSD-like behavior in mice. In line with our results, as the critical protein of the ECS, MAGL expression increases at both the mRNA and protein levels, thereby increasing anxiety-like behaviors during acute and chronic stress (Guggenhuber et al., 2015). Nevertheless, using RNA interference to knock down the expression of MAGL reduces anxiety-like behaviors (Lomazzo et al., 2015). Similarly, an inhibitor of FAAH rescues anxiety phenotype, supporting the notion that eCB deficiency plays a pathophysiological role in the brain (Lutz et al., 2015). Additionally, as the major protein for the synthesis of eCB, loss of DAGLα facilitates anxiety in mice (Jenniches and Zimmer, 2016). Based on these findings, we speculate that it is possible that regulating gene expression levels represents a way for ZZX to compensate for eCB in PTSD mice.

In line with these results, a treatment of traditional Chinese medicine with ZZX ameliorates PTSD-like behaviors in mice. It is noted that SSRI inhibits the reuptake of 5-HT by the releasing site, and paroxetine, a drug approved by the FDA for PTSD, has been shown to promote the level of 5-HT in the body to increase the antidepression action (Lawford et al., 2003). Numerous studies have shown that ZZX treatment, as well as the administration of other traditional Chinese medicine, is beneficial in significantly lowering depression and anxiety scores in patients, although the relative efficacy of traditional Chinese medicine for antidepression compared to SSRIs is still a matter of debate. ZZX in particular was recently shown to alleviate stress and anxiety. The positive influence of ZZX administration on mood may rely on multiple mechanisms, including the regulation of eCB production, HPA axis, and neuromodulation. Additionally, based on the results of network pharmacology, the potential participation of other neurobiological factors has not yet been revealed and may be involved in immunomodulation, inflammatory mediator, cytoprotective signal pathways, and calcium homeostasis.

In the present study, we have explored the mechanisms by which traumatic stress and contextual fear memory contribute to brain dysfunctions and behavioral abnormalities associated with PTSD-like states. Especially, the mechanisms may involve subtle changes of the neurotransmitters, alterations of the HPA, or changes in eCB metabolic pathways. Despite increasing evidence that PTSD is associated with decreased quality of life, increased depression, and chronic stress, there are currently no mechanism-based treatments available. Searching for mechanistic explanations of these dysfunctions, we show that loss of eCB provides a potential explanation for increased PTSD in mice and that targeting the ECS may promise as a therapeutic strategy. Altogether, these results also prompted us to consider and search for effective new therapeutic targets for PTSD. In addition to these possible pathways of action, the potential participation of other neurobiological factors cannot be ruled out, such as the body’s immunomodulation and inflammatory mediators; the activation of cytoprotective signal pathways, such as protein kinases and neurotrophic factors; the modulation of cell excitability and calcium homeostasis *via* alterations in Ca^2+^, K^+^, and Na^+^ channels, NMDA receptors, and intracellular Ca^2+^ stores. Thus, further studies should be conducted to test this possibility by using inhibitors or gene knockouts. While our study shows that ZZX plays a role in anti-PTSD, our experimental strategy was not capable of distinguishing which components are responsible (Friesner et al., 2006 Galanopoulos et al., 2014 Monte et al., 2013 Yu et al., 2016.

## Conclusion

ZZX plays an essential role in preventing PTSD. From a mechanistic perspective, our results suggest that ZZX may provide new therapeutic pathways for treating PTSD by the regulation of neurotransmitters, the HPA, and the ECS in the brain (Tables 3–5).

**TABLE 3:** Regression equations and correlation coefficients of five components in ZZX extract.

Component	Regression equation	Correlation coefficient	Linearity range (μg/ml)
Valtrate	y = 10907x – 25102	0.9996	18–450
Acevaltrate	y = 13534x – 260128	0.9996	30–720
Homobaldrinal	y = 30494x + 28059	0.9996	10–260
Baldrinal	y = 9894.8x + 1785.1	0.9996	0.5–25
11-ethoxyviburtinal	y = 22113x + 96281	0.9997	11–197

**TABLE 4:** Contents of five components in ZZX extract.

Compound	Retention time (min)	Contents (mg/g)
Valtrate	26.11	8.845
Acevaltrate	23.75	13.361
Homobaldrinal	11.21	0.606
Baldrinal	9.16	0.500
11-ethoxyviburtinal	7.34	0.489

**TABLE 5:** Reflection of clinical symptoms of PTSD in the DSM-5 phenotypic model.

Clinical symptoms of PTSD	Anhedonia model	Externalize behavioral models	Hybrid model
B1. Intrusion	I	I	I
B2. Nightmare	I	I	I
B3. Flash back	I	I	I
B4. Emotional response	I	I	I
B5. Physiological response	I	I	I
C1. Avoidance of trauma-related thoughts	A	A	A
C2. Avoidance of traumatic cue	A	A	A
D1. Amnesia of trauma-related	NA	NACM	NA
D2. Negative belief	NA	NACM	NA
D3. Distortion and blame	NA	NACM	NA
D4. Persistent negative emotional state	NA	NACM	NA
D5. Loss of interest	An	NACM	An
D6. Emotional alienation	An	NACM	An
D7. Inability to experience positive emotions	An	NACM	An
E1. Irritability or indignation	DA	EB	EB
E2. Reckless or self-destructive behavior	DA	EB	EB
E3. Hypervigilance	AA	AA	AA
E4. Startle reaction	AA	AA	AA
E5. Attention problems	DA	DA	DA
E6. Sleep problems	DA	DA	DA

I, intrusion; A, avoidance; NACM, negative cognitive and emotional changes; DA, mental distress arousal; AA, anxiety arousal; NA, negative emotions; An, anhedonia; EB, externalized behaviors.

## Data Availability

The original contributions presented in the study are included in the article/Supplementary Material, and further inquiries can be directed to the corresponding authors.
