# Supplementary figures and images for: Valeriana jatamansi Jones ex Roxb. Against Post-Traumatic Stress Disorder, Network Pharmacological Analysis, and In Vivo Evaluation
Source: Front Pharmacol. 2021 Dec 7;12:764548. doi: 10.3389/fphar.2021.764548 (PMC8688958; doi:10.3389/fphar.2021.764548)

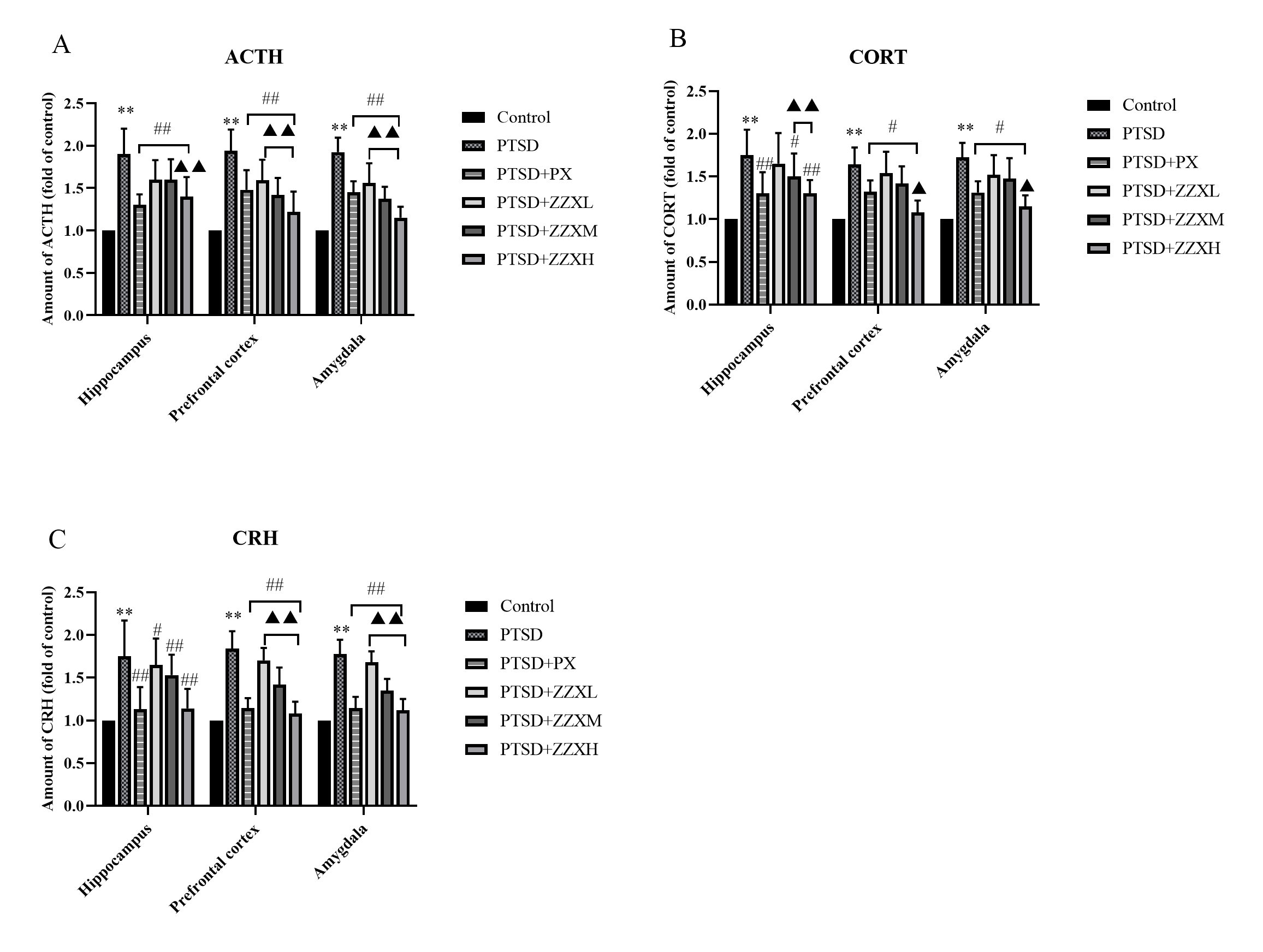

Supplement: Supplementary file 1 [file Image3.TIF]

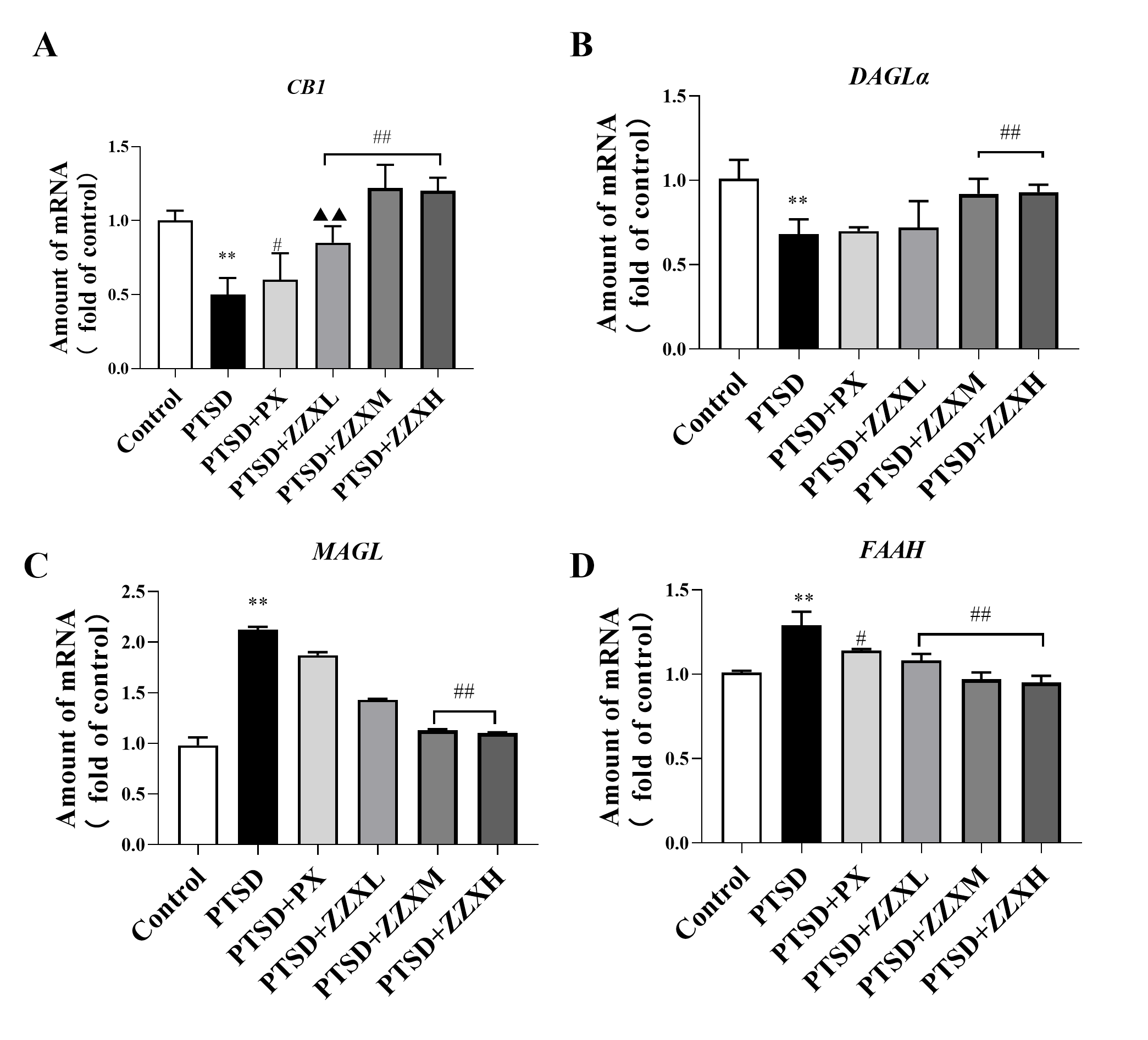

Supplement: Supplementary file 2 [file Image4.TIF]

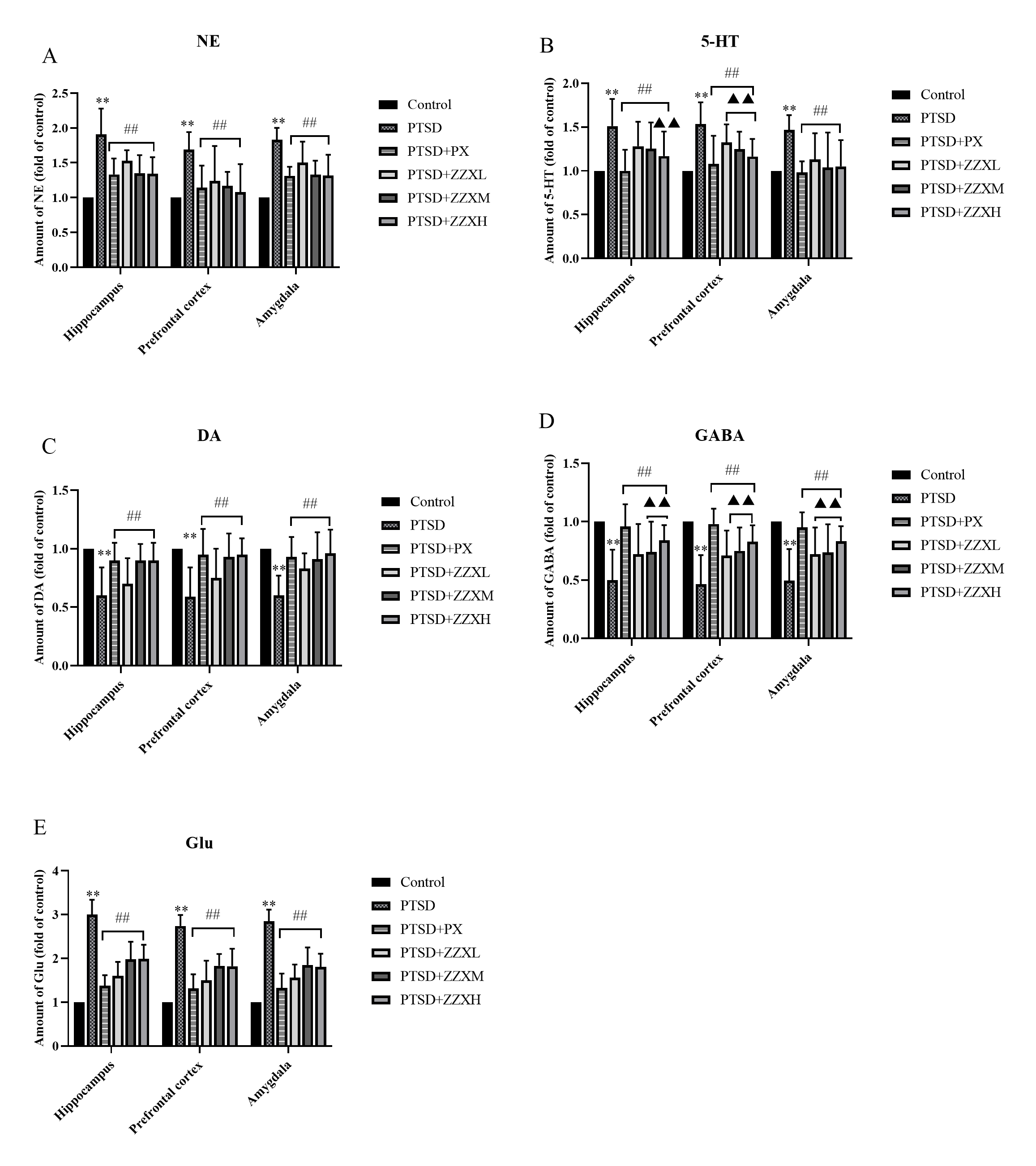

Supplement: Supplementary file 3 [file Image2.TIF]

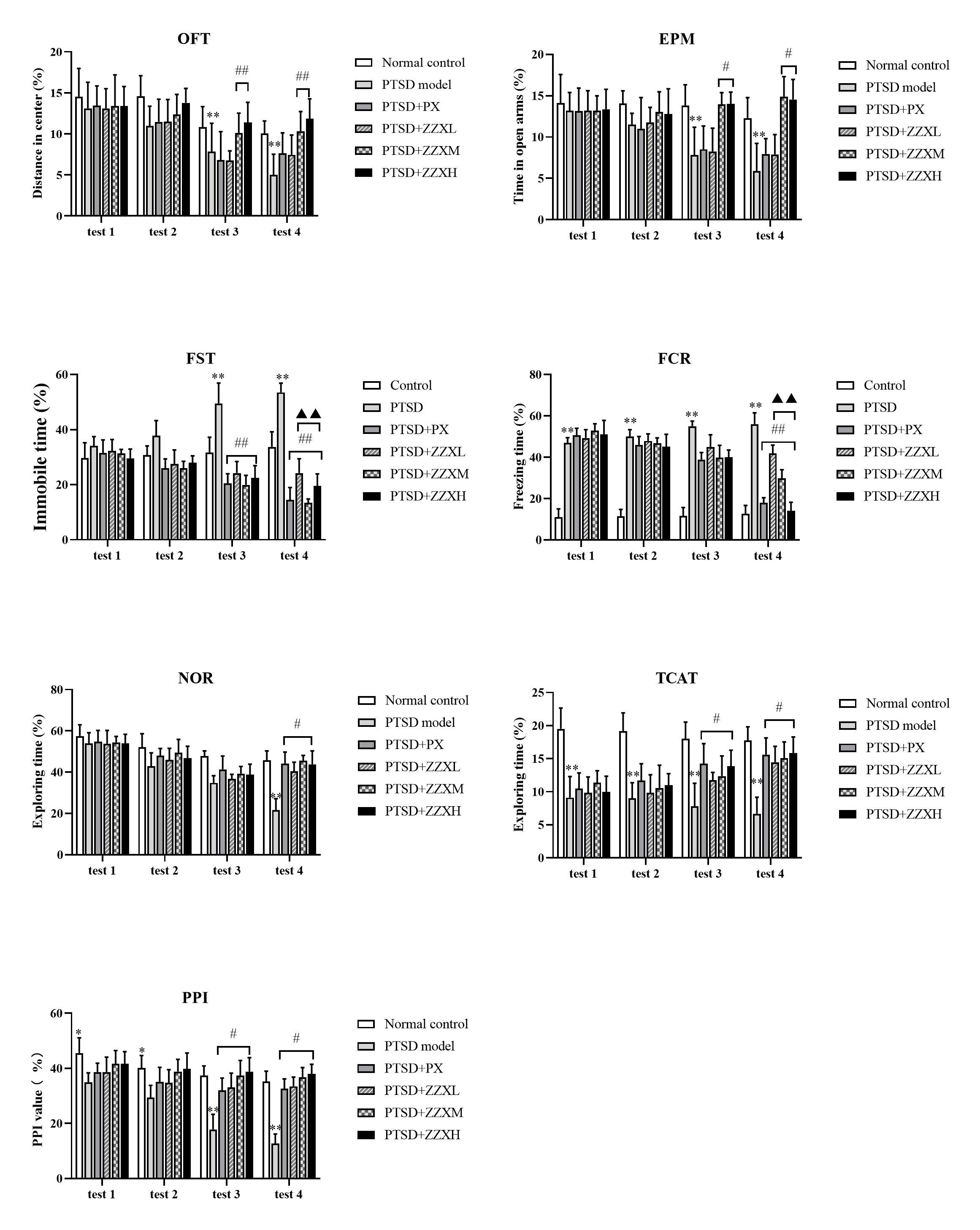

Supplement: Supplementary file 4 [file Image1.TIF]
